# Subcutaneous Injections of Iron

**Published:** 1891-11

**Authors:** 


### SUBCUTANEOUS INJECTIONS OF IRON.

Dr. Rosenthal has made an interesting study of the action of subcutaneous injections of iron in nervous affections. By personal experiment he ascertained that when a preparation of iron was administered subcutaneously, it was absorbed thirty to forty minutes after injection, and could be detected in the urine. From the basis of a long series of investigations, the author recommends two iron preparations for hypodermic injection. One is the so-called ferrum peptonatum, obtained by the decomposition of ferric chloride solution with solution of pepsin, as a brownish-yellow powder, readily soluble in water. It is applied in aqueous solution (1 to 10), one syringeful being given every second day. The second suitable preparation is ferrum oleatum, diluted to one to twenty by olive oil, and used similarly. Of the two, the former is preferable, on account of its greater solubility and stability. The subcutaneous iron treatment is recommended by Rosenthal in delicate neurasthenic persons, and in the asthenic dyspepsia, so often associated with anemia, in which small doses of iron may produce considerable digestive disturbance. Unpleasant by-effects did not appear.—*Provincial Medical Journal*, September 1, 1891.—*Therapeutic Gazette.*

